# Validation of a brain MRI relaxometry protocol to measure effects of preterm birth at a flexible postnatal age

**DOI:** 10.1186/1471-2431-14-84

**Published:** 2014-03-28

**Authors:** Nathalie L Maitre, James C Slaughter, Ann R Stark, Judy L Aschner, Adam W Anderson

**Affiliations:** 1 Department of Pediatrics, Vanderbilt University, Nashville, TN, USA; 2 Department of Biostatistics, Vanderbilt University, Nashville, TN, USA; 3 Department of Pediatrics, Albert Einstein College of Medicine, Bronx, NY, USA; 4 Departments of Biomedical Engineering and of Radiology and Radiological Sciences, Vanderbilt University, Nashville, TN, USA; 5 Division of Neonatology, The Monroe Carell Jr. Children’s Hospital at Vanderbilt 11111 Doctor’s Office Tower, 2200 Children’s Way, Nashville, TN 37232-9544, USA

**Keywords:** MRI, Relaxometry, Basal ganglia, Globus pallidus, Maturation, Prematurity, Neonate

## Abstract

**Background:**

Magnetic resonance imaging (MRI) is a useful tool to study brain growth and organization in preterm neonates for clinical and research purposes, but its practicality can be limited by time and medical constraints. The aim of this study was to determine if MRI relaxometry of the deep nuclei, as opposed to white matter, would reflect the influence of gestational age at birth on structures essential to motor development, regardless of postnatal age at the time of imaging.

**Results:**

This was a prospective observational study of infants without brain injury on conventional neuroimaging who were cared for in the neonatal intensive care unit (NICU) at Vanderbilt. Infants were studied using MRI relaxometry within a 2-month window of postmenstrual term age. In 45 infants, white matter MRI T1 relaxation times were influenced by both gestational age and postnatal age at imaging time (R^2^ = 0.19 for gestational age vs. R^2^ = 0.34 adjusting for both gestational age and age at imaging; all p < 0.01). Similar results were obtained with T2 relaxation times. In contrast, globus pallidus T1 reflected gestational age but was minimally affected by postnatal age (R^2^ = 0.50 vs. 0.57, p < 0.001).

**Conclusions:**

The results obtained using this imaging protocol are consistent with the slow maturation of the globus pallidus, essential to normal development of complex motor programs into adulthood. Globus pallidus MRI relaxometry measures the impact of gestational age at birth on brain development independent of postnatal age in preterm infants and should prove useful for predictive modeling in a flexible time-window around postmenstrual term age.

## Background

Infants cared for in neonatal intensive care units (NICUs) are at increased risk for abnormal brain development and contribute disproportionately to the burden of developmental disabilities [1-5]. Interruption of normal development imposed by premature birth can be magnified by disruptions in neurologic development caused by environmental toxins, medical insults and nutritional deficits [6,7].

Neuroimaging is an attractive tool for assessing environmental influences on neonatal brain growth and organization as it is non-invasive and has direct anatomic specificity. While ultrasound is routinely used for diagnostic and counseling purposes, [8,9] magnetic resonance imaging (MRI) using conventional techniques offers a quantitative advantage [10-15]. Magnetic resonance (MR) relaxometry, which measures the time it takes for nuclear spins to return to thermal equilibrium after being disturbed, conveys information about the molecular environment of water in tissues. The time constants for relaxation parallel and perpendicular to the polarizing magnetic field (T1 and T2, respectively) depend on different aspects of spin motion and exchange; [16] they are therefore sensitive to changes in the lipid and water content of tissues. Hence, MR relaxometry provides a quantitative measure of central nervous system (CNS) organization [17] that can be incorporated into predictive models of brain development, to inform clinicians and researchers on the consequences of the infant’s condition and intensive care course.

Imaging of former preterm infants for assessment of neurologic injury is typically performed within a narrow time-window near term postmenstrual age because postnatal age and maturation may differentially affect MRI indices. However, this timing is not always practical. An MRI relaxometry paradigm to measure brain maturity during a wider window of time around term-equivalent age might facilitate the development of predictive models of neurodevelopment before hospital discharge. We focused on the deep nuclei of the brain because these structures are less affected by myelination than white matter regions. The lesser influence of postnatal age on nuclei, such as the globus pallidus, is consistent with a maturation process that continues into early adulthood: this slow progression is essential to establish complex motor programs regulating the relatively simpler movements performed via myelinated tracts [18].

Our goal, therefore, was to use MR relaxometry to develop quantitative methods for measuring the effects of preterm birth on deep nuclei. We hypothesized that, in infants without overt neural injury, relaxometry indices in deep nuclei would reflect changes associated with gestational age at birth (GA) but be less influenced by postnatal age at MRI than white matter tracts.

## Methods

### Study design and subjects

This methodological development study made use of a subset of 52 infants with MRI performed within 2 months of term equivalent (defined as 40 weeks postmenstrual age). These infants were part of a prospective, observational study of preterm and term infants at the Monroe Carell Jr. Children’s Hospital at Vanderbilt in Nashville, TN between November 2004 and July 2010. Infants in the current subset were recruited from the Vanderbilt NICU. The goal of the larger study was an observation of common nutritional exposures and included older children born at term who were not part of the current cohort, and were recruited from the Children’s Hospital pediatric radiology outpatient schedule. Ethical approval for this research was obtained from the Vanderbilt Institutional Review Board (protocol #07077 and 04881). All parents of participating infants signed Vanderbilt IRB-approved informed consent documents prior to MRI. Excluded infants were those not expected to survive to the age of 3 months, intubated infants whose clinical instability increased the risks associated with transport from the NICU for the MRI procedure or those with a confirmed diagnosis of hypoxic- ischemic encephalopathy. A pediatric neuroradiologist reviewed clinically indicated MRIs to exclude those with white matter injury or lesions in the deep nuclei.

Gestational age at birth was ascertained from the best obstetric estimate, based on the mother’s last menstrual period, obstetric measurements, and ultrasound measurements taken in the first trimester of pregnancy [19]. Clinical data were obtained from the medical record.

### Image acquisition

Subjects were placed in a MRI compatible papoose (Universal Medical, Norwood, MA) to minimize movement artifact following published recommendations [20] and scanned on a Philips Achieva 1.5 T MRI scanner (Philips, Amsterdam, Netherlands). Infants were swaddled and fed prior to MRI when possible. Those who fell asleep were scanned without sedation. Those who were fussy received oral chloral hydrate. None of the infants were intubated or given narcotics for the purposes of the study scan. T2-weighted images (dual echo, turbo spin echo, TR = 3 s, TE = 16.8 and 210 ms, in-plane resolution 0.7 × 0.7 mm, number of slices = 18, slice thickness = 4 mm, slice gap = 1 mm, number of acquisitions = 3, echo train length = 16, scan time = 6:06) and T1-weighted images (inversion-prepped turbo spin echo, TR = 8.3 s, TE = 15 ms, in-plane resolution. 0.7 × 0.7 mm, number of slices = 18, slice thickness = 4 mm, slice gap = 1 mm, number of acquisitions = 2, echo train length = 16, scan time = 3:03, inversion delays T1 = 300, 500, 700, 1000, 1500, 2000, and 2500 ms) were acquired. Images were oriented in coronal planes for all subjects.

### Image analysis

Tissue T2 relaxation time was calculated for each image voxel, assuming monoexponential signal decay. Tissue T1 was calculated using a two-parameter fit for T1 relaxation time and equilibrium magnetization. First, complex image data were phase-corrected using one of the scans (usually the scan with the shortest inversion delay) as a phase reference. The signal amplitude was then fit to a standard inversion recovery function [21]. Ideally, each inversion delay time (T1) contributed one data point to the fit. However, in some cases, subject motion corrupted the images, producing strong ghost artifacts. The entire image volume was discarded when motion-induced ghost artifacts corrupted the images. If the number of time points with uncorrupted data fell below four, no T1 fit was attempted. All calculations were performed using the MATLAB (The Mathworks, Natick, MA) programming language.

**Figure 1:**
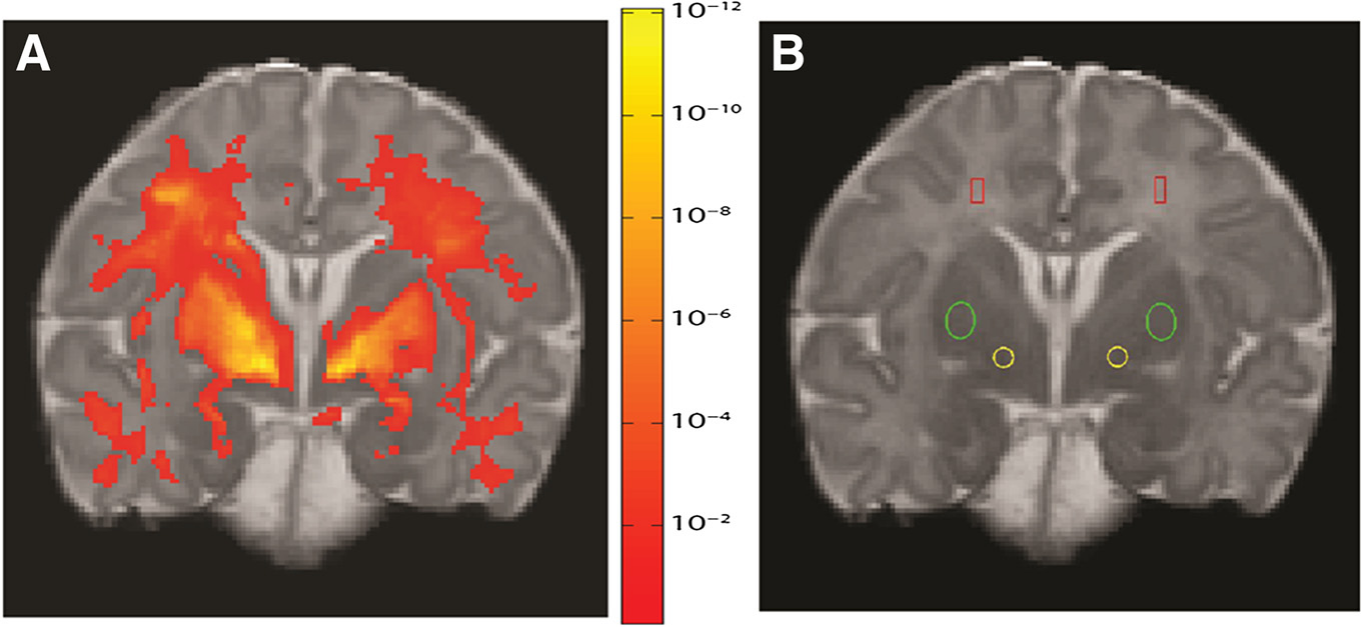
**Reference brain section with regions of interest and associations. A**: Association between gestational age and T1 relaxation time across the study population. Color indicates the statistical significance (p value) of voxel-wise correlations. Voxels were thresholded at the p < 0.001 level and permutation testing was used to control false positive errors at the cluster level (p < 0.01). **B**: Regions of interest defined in a reference section (Red = Centrum Semiovale, Green = Putamen, Yellow = Globus Pallidus Internus).

Maps of T1 and T2 relaxation times were aligned in a common space to improve the accuracy of comparisons across subjects. One subject (Figure 1A) was chosen to define a reference set of brain images (the remaining T1 and T2 data for this subject were discarded to avoid bias in the final result). The T1 and T2 maps for other subjects were spatially registered to this reference using linear transformations (i.e., translation, rotation, scaling, and shearing in three dimensions) and maximizing mutual information [22]. We employed a standard affine registration algorithm optimizing normalized mutual information and using three spatial resolution levels [22]. We chose not to smooth the registered image data (beyond the smoothing inherent in interpolation) in order to avoid partial volume averaging/contamination from neighboring structures. T2 maps were aligned to the reference brain’s T1 data by finding the linear spatial transformation that maximized the normalized mutual information between the two data sets.

Regions of interest (ROIs) were defined in the globus pallidus internus (GP), putamen (PN) and centrum semiovale (CS) in both hemispheres of the reference brain image set (Figure 1B). Areas were chosen to have no overlap of grey and white matter in the reference brain and in the neuroanatomical references [23]. Choice of GP and PN ROIs was motivated by the important role these play in motor function and implicit learning, functions that undergo rapid development in the postnatal period. Choice of GP also motivated the use of coronal section for clearest possible definition. Choice of CS was directed by need for white matter representation as related to motor function, as well as inter-hemispheric connectivity, without the drawbacks of using vulnerable areas in the direct periventricular region. The net areas of the ROIs were 13 mm^2^ (GP), 27 mm^2^ (PN) and 12 mm^2^ (CS). When overlaid on the transformed T1 and T2 maps for other subjects, the ROIs in some cases revealed small displacements relative to the intended structure, due to limitations of linear transformations between brain images. These displacements were corrected by shifting the ROIs slightly along the image row and/or column direction so they became centered on the structure of interest. The mean relaxation time (T1 or T2) was calculated for each region and subject.

In addition to the region-of-interest analysis, a univariate linear regression was used in voxel-by-voxel tests for the effects of GA on T1 relaxation time:

$$T1=a_{0}+a_{1}*\mathrm{GA}$$

This analysis was performed in the common space described above. Voxels were thresholded at the p < 0.001 level and permutation testing [24] was used to control false positive errors at the cluster level (p < 0.01).

### Statistical analysis

Separate linear regression models were used to estimate the association of gestational age and days to MRI with T1 and T2 at each ROI. Univariable and multivariable models were estimated and summarized by the R^2^, Akaike Information Criterion (AIC), and, for univariable models, the statistical significance of the predictor. R^2^ summarized the percentage of the variability in the outcome explained by the predictors in the model. The AIC is a method of model selection that allowed for comparing non-nested models, in this case, comparing a model with GA to a model with days to MRI, while penalizing for model complexity. AIC values were used to compare these models with different predictors but the same outcome, with lower values of AIC indicating a better model fit.

## Results

### Subject demographics

Of the initial sample of 52 patients, 7 were excluded due to evidence of either white matter injury or lesions in the deep nuclei, leaving a normative sample of 45 patients for the study. Three of the 45 study subjects were full term infants recruited from the Children’s Hospital rather than from the NICU. Study infant characteristics are shown in Table 1. The infant subjects were born at gestational ages representing the full spectrum of early preterm to full term birth (24–40 weeks). The median number of days of age at MRI was 49, corresponding to a median corrected GA of 38 weeks at testing (interquartile range 34–41 weeks).To confirm the choice of regions of interest for the quantitative analysis, we compared the statistical parametric map showing the voxel-wise dependence of T1 on GA to the regions of interest. The effect of GA on T1 values in the coronal slice containing the standard ROIs can be visualized using a map of p values for the dependence on GA, where higher significance appears in warmer (redder) colors (Figure 1A). This demonstrates that the strongest effect of GA appears in the white matter, PN, and GP.

**Table 1:** Study population characteristics (N = 45)

	**N**	**Descriptives**
**Infant characteristics**		
GA at birth, *Median (IQR) in weeks*	45	31 (26, 37)
PMA at MRI, *Median (IQR) in weeks*	45	38 (34, 41)
Postnatal age at MRI, *Median (IQR) in days*	45	49 (25, 91)
Sex female, *N (%)*	45	23 (51)
**ROI values (ms**^**-1**^**)**		
GP, *Median (IQR)*		
T1	45	1146 (1075,1251)
T2	42	168 (158,180)
PN, *Median (IQR)*		
T1	45	1250 (1141,1350)
T2	42	168 (155,178)
CS, *Median (IQR)*		
T1	44	2027 (1751,2154)
T2	41	323 (282,369)

SD = standard deviation.

IQR = interquartile range, 25th to 75th percentile.

GA = gestational age.

PMA = postmenstrual age.

**Figure 2:**
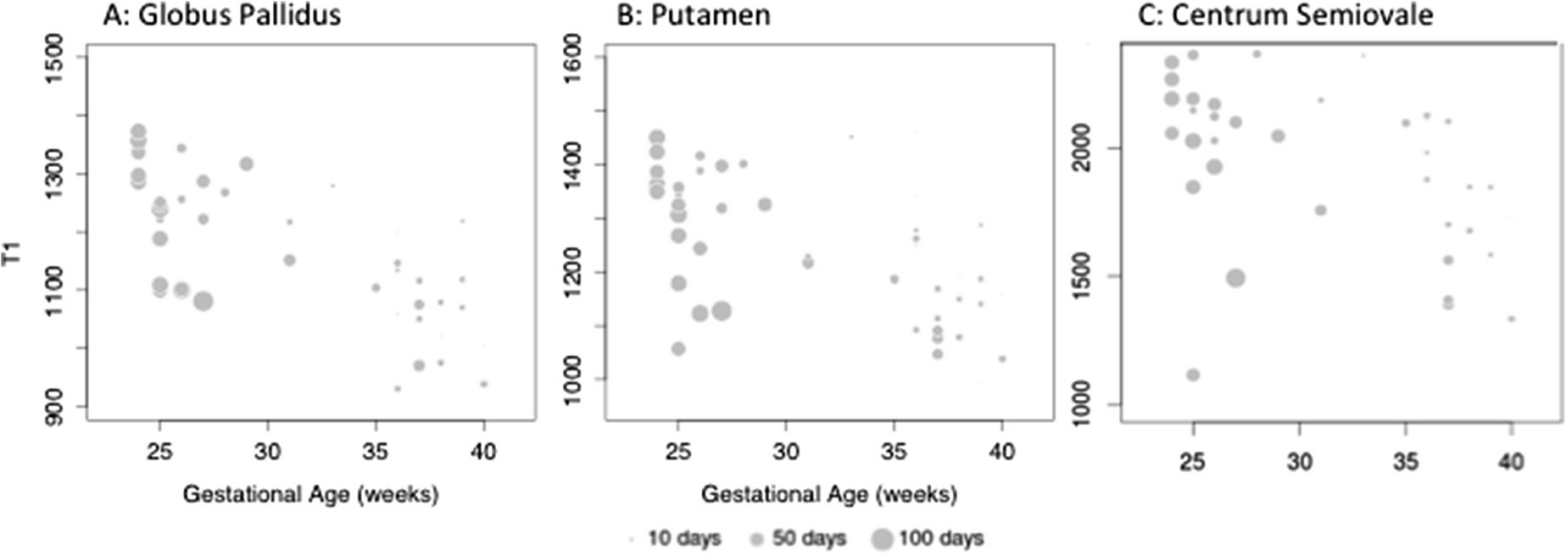
**Relationship between T1 indices, gestational age at birth and age at MRI.** T1 indices are measured on the Y-axis and GA at birth on the X-axis. For all three panels, the size of the circle represents the number of days between birth and MRI. Each panel shows the results of separate ROIs: **Panel A**: Globus Pallidus; **Panel B**: Putamen; **Panel C**: Centrum Semiovale.

To develop a quantitative model for clinical use, we then focused on the defined ROIs in the GP, PN as well as the CS as a comparison for myelinated tissue (Figure 1B). For all ROI and all indices except GP T2, the R^2^ for univariable models including GA were much larger than the R^2^ for univariable models containing days to MRI. In these same regions, the AIC was also lower for the models with GA than the models with days to MRI, indicating that the GA models had better overall fit (Table 2). The multivariable models for PN and CS had both higher R^2^ and lower AIC than the unadjusted models. The exception was GP T1 in which the multivariable model was similar to the univariable model using GA as a predictor (R^2^ 0.57 AIC 532 vs. R^2^ 0.50 AIC 538). As gestational age at birth increased, T1 in the GP, PN and CS decreased, with age at MRI having a much smaller influence in the GP than in the PN or CS. These relationships are illustrated in Figure 2 (A-C), where T1 is plotted as a function of GA, with circle size representing age at MRI.

**Table 2:** Effect of GA on relaxometry indices

**ROI**	**Indices**	**Gestational age (unadjusted)**			**Days to MRI (unadjusted)**			**Multivariable**	
		**R**^**2**^	**AIC**	**p-value**	**R**^**2**^	**AIC**	**p-value**	**R**^**2**^	**AIC**
GP	T1	0.50	538	<0.001	0.15	561	0.01	0.57	532
	T2	0.01	442	0.54	0.08	439	0.07	0.12	440
PN	T1	0.31	554	<0.001	0.02	570	0.35	0.54	538
	T2	0.21	351	0.003	0.01	360	0.7	0.68	314
CS	T1	0.19	645	0.003	0.01	654	0.49	0.34	638
	T2	0.16	462	0.01	0.02	469	0.89	0.46	446

AIC: Akaike Information Criterion.

## Discussion

Our study demonstrates that MR relaxometry indices in the GP and the CS can provide a quantitative assessment of the effects of gestational age and postnatal age on the brains of preterm and term neonates.

While white matter is a natural choice to study the level of maturation and organization of the neonatal brain, [25] recent studies have suggested that deep nuclei and grey matter may also reflect the level of neural and organizational disruption in preterm and term infants. For example, magnetization transfer ratios in the basal ganglia can reflect gestational age at scan time [26]. Tissue volume reductions in the GP can be combined with measures of periventricular white matter structure to provide a more complete picture of diffuse injury that correlates well with global developmental scores in early childhood [27,28].

Importantly, the relationship between GP T1 and GA appears minimally affected by postnatal age within a 2-month window of term-equivalent age. Clinically, this is consistent with the expected maturation of a structure that continues to play an essential role in the coordination of motor functions into late adolescence. As in other studies of preterm infants, we showed that CS T1 and T2 measures depend on both degree of immaturity at birth and the maturation that occurs in the neonatal period [25].

The effects of GA are specifically observed in GP T1 but not T2 indices. Any interpretation of the difference between T1 and T2 relationships remains speculative, but presumably the spin-spin interactions underlying T2 are dominated by tissue constituents that are not changing significantly during this period [29]. It is possible that T2 changes are not significant in the GP due to the origin, relative maturity and composition (e.g., paucity of myelinated fibers) of this structure compared to the putamen. In particular, the primarily diencephalic instead of telencephalic origin of the GP, along with evidence of synaptogenic maturity as early as 15 weeks GA, is in sharp contrast to the putamen (telencephalic origin) with synaptogenic maturity and microstructure emerging at 22 weeks GA. The difference in T1 and T2 patterns in the GP may therefore reflect maturation independent of early embryonic changes [30].

In contrast to the effects of GA on GP T1, postnatal age at MRI has a far smaller influence, especially when comparing effects on the PN or the CS. This may reflect the paucity of white matter tracts and changes in myelination in our GP ROIs. This relative stability in the face of neonatal development can present advantages for research in the NICU. The medical conditions of NICU infants combined with hospitalizations that vary from weeks to months can preclude narrow time intervals for imaging in longitudinal studies. The use of GP T1 relaxation times would therefore allow a quantitative and flexible measure of an exposure’s effects while still accounting for neuroimaging differences caused by prematurity. This would be particularly relevant to the assessment of nutritional exposures shown to result in neurotoxicity or degeneration in adults, such as manganese, iron or copper [31]. The requirements and metabolism of paramagnetic nutrients such as iron or manganese, [32] is of special concern in preterm populations at risk for either deficits or excess of these elements [33,34]. These trace elements could be quantified and the resulting exposure correlated with changes in the basal ganglia before NICU discharge and potentially with subsequent neurodevelopmental outcomes.

As in other studies of preterm infants, we showed that T1 and T2 measures of white matter depend on both the degree of immaturity at birth as well as the maturation that occurs in the neonatal period [35]. Our choice of the CS for white matter avoids those areas most susceptible to injury in the preterm brain, such as the internal capsule and periventricular white matter, [36] and has the added advantage of slow maturation in the first year of life [37]. CS fibers constitute an essential component of corticospinal tracts [37] and are associated with motor development in early childhood [38,39]. Fractional anisotropy of the white matter has shown that organization of the CS also reflects white matter injury and later motor impairments [40,41].

There are a number of limitations to the MRI measurements. First, in order to limit scan time, the slice thickness was set to 4 mm (with an inter-slice gap of 1 mm). This makes spatial resolution in the through-slice direction relatively coarse, which may reduce inter-subject registration accuracy in the through-slice direction. We also excluded infants with overt injury as determined by neuroradiologists using conventional clinical imaging protocols. This does not account for possible injury as visualized with research protocols using diffusion-weighted imaging techniques, for example. Our choice to omit this type of imaging was motivated by the need to acquire reliable estimates of T1 without lengthy image acquisition protocols. Finally, our statistical models tested for the effects of gestational and postnatal age, but did not test for other uncontrolled factors that could contribute to MRI relaxivity in the brain.

Finally, several systematic errors can affect T1 and T2 estimates [42]. Practical measurements usually represent a compromise between imaging time and accuracy. In order to minimize scan time, our measurements employed multiple echo pulse sequences, which are influenced by implementation details that may differ between scanner manufacturers or even between hardware and software versions provided by the same manufacturer. Therefore, it is likely that the same subjects scanned on other equipment would yield slightly different mean T1 and T2 values. System-dependent bias in relaxation times is expected to be nearly the same for all subjects in the study, however. Hence the dependence of T1 and T2 on gestational age and postnatal age are expected to be system-independent and our results should be reproducible on other scanner platforms.

## Conclusion

The relative stability of GP T1 in the face of postnatal development can be advantageous for the development of neuroimaging protocols predictive of outcomes in neonatal intensive care infants. The medical conditions of these patients and the variability of their hospitalizations from weeks to months can preclude narrow time-intervals for imaging studies, reducing the feasibility of MRI for research, especially in institutions not specifically prepared for neonatal imaging. The use of GP T1 may therefore allow a quantitative and flexible measure of an intervention or exposure’s effects on neuroanatomic microstructure disruptions caused by prematurity.

## Abbreviations

MRI: Magnetic resonance imaging; NICU: Neonatal intensive care unit; MR: Magnetic resonance; CNS: Central nervous system; GA: Gestational age at birth; ROIs: Regions of interest; GP: Pallidus internus; PN: Putamen; CS: Centrum semiovale; AIC: Akaike information criterion.

## Competing interests

The authors declare that they have no competing interests.

## Authors’ contributions

NM formulated the hypothesis, defined the ROIs, designed the analysis, collected the data and wrote all drafts of the manuscript. JS performed and revised the statistical analysis and edited all drafts of the manuscript. AS participated in the formulation of the hypothesis, refining of the results and edited all drafts of the manuscript. JA conceived of the larger studies and obtained funding for them, participated in the design of the current study, and helped edit all drafts of the manuscript. AA carried out the MRIs, designed the analysis of MRI all MRI data, addressed and revised all versions of the manuscript. All authors read and approved the final manuscript.

## Pre-publication history

The pre-publication history for this paper can be accessed here:

http://www.biomedcentral.com/1471-2431/14/84/prepub
